# Control of hyperhydricity of *Pistacia khinjuk* stocks in vitro shoots

**DOI:** 10.1186/s12896-024-00929-3

**Published:** 2024-11-28

**Authors:** Yusuf Ersali

**Affiliations:** Department of Food Processing, Vocational School of Technical Science, Batman University, Batman, Turkey

**Keywords:** Ammonium nitrate, Cytokinin, Hyperhydricity, MS, Shoot

## Abstract

Hyperhydricity is the most extensive physiological disorder during in vitro propagation. This disturbance can induce anatomical, morphological and physiological problems that cause serious damage. The factors that cause hyperhydricity are the composition of nutrient media and cultures conditions. To reduce the hyperhydricity of *Pistacia khinjuk*, ammonium nitrate (NH_4_NO_3_), calcium chloride (CaCl_2_·2H_2_O), cytokinins of *meta*-topolin (*m*T) and 6-benzylaminopurine (BAP) at different concentrations were investigated in Murashige and Skoog (MS) medium. The lowest percentage of hyperhydricity (34.30%) were obtained from the medium containing 1650 mg/L NH_4_NO_3_, 110 mg/L CaCl_2_·2H_2_O and1 mg/L *m*T; the highest percentage of hyperhydricity (68.42%) were obtained from the medium containing 206.25 mg/L NH_4_NO_3_, 440 mg/L CaCl_2_·2H_2_O and 0.5 mg/L BAP. The maximum average number of shoots per explant (2.45), average shoots length (18.47 mm) and proliferation rate (85%) were obtained from the medium containing 1650 mg/L NH4NO3, 110 mg/L CaCl2·2H2O of MS and 1 mg/L mT. In addition, when soluble protein (2.12 mg/g) and total chlorophyll a, b (0.96 mg/g) value of normal (non-hyperhydric) shoots were higher than hyperhydric shoots, carotenoid (11.75 µg /g) and water content (78.70%) value of normal shoots were lower than hyperhydric shoots. This study concludes that the hyperhydricity percentage of *in vitro P. khinjuk* shoots was reduced (12.8%) on modified MS medium with NH_4_NO_3_, CaCl_2_·2H_2_O and *m*T according to standard MS medium.

## Introduction

Pistachio (*Pistacia vera* L.) is used in sweets, ice cream, or consumed fresh [1] and had an annual gross value of 6 billion dollars in the year of 2022–2023 [2]. *Pistacia khinjuk* Stocks is one of rootstocks of pistachio, and they are traditionally propagated by seeds. However, germination rates are too low (15–25%) [3] for commercial propagation and this is the main problem for the establishment of new pistachio gardens [4]. Since these rootstocks cuttings are not rooted as successfully as in other tree species [5], new propagation methods are being explored for commercializing pistachios, including in vitro propagation. This biotechnological process makes it possible to reproduce genetically superior and disease-free woody plant species in a limited time and space. However, the method of in vitro propagation has some problems such as low proliferation rates and hyperhydricity [6], which come from high humidity, excess nitrates, lighting, and plant growth regulators [7]. In vitro propagation of *P. khinjuk* [8, 9] and *P. vera* [10] species was successfully done but hyperhydricity was not investigated in these studies. Hyperhydricity is the most common physiological defect in micropropagated plants [11]. Hyperhydric (glassy, vitrify) tissues have semitransparent and fragile, thickened, short internodes stem and translucent, elongated, and twisted leaves [11]. These plants have chlorophyll defects, surplus fluid in the intercellular spaces, and changes in protein synthesis due to the deterioration of typical metabolic processes [12]. Defects are expressed mostly on leaves, but also stems and roots. Hyperhydricity has seriously negative affects on micropropagation performance most micropropagated plant species [13]. Since propagation capacity of hyperhydric shoots is low and most hyperhydric tisseus die eventually in culture medium [14]. Hence these plants have poor survival rates in *ex vitro* conditions. Despite continuing research on in vitro pistachio cultures, they still have low proliferation rates, shoot tip necrosis, leaf necrosis, excessive callus growth, and hyperhydricity [6]. A recent study on shoot proliferation of *P. vera* L. found that the lowest hyperhydricity rate was 59% [15]. Various concentrations and combinations of ammonium nitrate (NH_4_NO_3_), calcium chloride (CaCl_2_·2H_2_O), *meta*-topolin (*m*T) cytokinins, and 6-benzylaminopurine (BAP) were tested. In addition hyperhydric and normal shoots (non-hyperhydric) were compared in terms of the soluble total protein, chlorophyll a, b, carotenoid and fresh/dry weight.

## Materials and methods

### Plant material and culture condition

*P. khinjuk* seeds were obtained from Batman Provincial Directorate of Agriculture and Forestry in November 2022. *P. khinjuk* trees were identified by Turkish Plants Data Service [16] in west of Raman Mountain (37°49’23”N, 41^°^08’01”E) Batman province in Türkiye. The fruits and coats were removed from the seeds to obtain kernels. The kernels were disinfected with 20% commercial sodium hypochloride for 20 min on a shaker at 200 rpm. Then, the seeds were washed three times with sterile distilled water before being placed in contact with 50 ml MS [17] in Magenta vessels (GA-7, Sigma Ltd.). The seed germination medium contains 6.5 g/L agar and without plant growth regulators and sucrose. Shoot tips or nodal bud segments obtained from the germinated seeds were proliferated in order to obtain stock cultures in standard MS medium containing 1 mg/L benzylaminopurine (BAP). All explants (shoot tips or nodal bud) that were used for the experimnts approxymately 1 cm long. All medium that were used for the determination of any parameter containing 30 g/L sucrose and 6.5 g/L agar and plant growth regulators. The pH of all medium was adjusted to 5.8 before autoclaving (120 °C for 20 min.). Plant growth regulators were added to the medium prior to adjustment of pH and sterilization. Cultures were incubated at 25 ± 2 °C under 16/8 h (Light/Dark) photoperiods with light conditions of 3500 lx created by white fuorescent light for 28 days. All experiments were repeated two or three times.

Calculation of hyperhydricity rate, average length of shoots, proliferation rate, average number of shoots and water content, analyses of soluble total protein, chlorophyll a, b and carotenoid were done instantly after incubation.

Standard MS medium was modified by different concentrations (Table 1) of NH_4_NO_3_ (1650, 825, 412.5 and 206.25 mg/L) and CaCl_2_·2H_2_O (440, 220, 110 and 55 mg/L). These concentrations were combined with each other.

**Table 1:** The effect of NH_4_NO_3_ and CaCl_2_ ·2H_2_O combinations under cytokinins on hyperhydricity of *P. khinjuk*

Hyperhydricity rate (%)							
		0.5 mg/L BAP*	1 mg/L BAP*	2 mg/L BAP*	0.5 mg/L mT*	1 mg/L mT*	2 mg/L mT*
NH_4_NO_3_ (mg/L)	CaCl_2_ ·2H_2_O (mg/L)						
**1650**	**440**	47.55 ± 0.25^ef^	43.70 ± 0.41^gh^	43.40 ± 0.30^h^	41.30 ± 0.20^g^	39.32 ± 0.38^e^	39.50 ± 0.23^c^
	**220**	47.95 ± 0.63^ef^	45.52 ± 0.49^g^	45.21 ± 0.47^g^	41.26 ± 0.63^g^	37.43 ± 0.23^e^	37.82 ± 0.18^f^
	**110**	44.42 ± 0.36^g^	43.35 ± 0.44^h^	41.53 ± 0.37^ı^	39.78 ± 0.64^h^	34.30 ± 0.62^e^	34.60 ± 0.51^g^
	**55**	45.28 ± 0.63^fg^	44.80 ± 0.40^g^	41.75 ± 0.25^ı^	40.90 ± 0.73^h^	36.45 ± 0.21^e^	37.34 ± 0.39^f^
**825**	**440**	52.45 ± 0.64^cd^	51.13 ± 0.23^e^	50.50 ± 0.51^f^	41.90 ± 0.20^g^	37.63 ± 0.18^e^	38.56 ± 0.73^f^
	**220**	51.40 ± 0.88^d^	49.40 ± 0.64^f^	50.90 ± 0.37^f^	42.62 ± 0.45^e^	37.42 ± 0.29^e^	38.39 ± 0.90^f^
	**110**	50.60 ± 0.86^de^	51.92 ± 0.20^e^	50.60 ± 0.30^f^	42.35 ± 0.53^f^	38.98 ± 0.47^d^	39.78 ± 0.70^e^
	**55**	50.80 ± 0.92^de^	52.50 ± 0.52^e^	51.80 ± 0.37^e^	43.21 ± 0.69^e^	39.70 ± 0.63^d^	39.62 ± 0.31^e^
**412.5**	**440**	55.15 ± 0.88^c^	55.12 ± 0.36^d^	53.30 ± 0.65^d^	45.25 ± 0.16^d^	39.81 ± 0.59^c^	41.70 ± 0.55^d^
	**220**	55.20 ± 1.22^c^	55.70 ± 0.58^d^	54.80 ± 0.52^c^	46.49 ± 0.32^d^	50.75 ± 0.43^c^	42.90 ± 0.75^bc^
	**110**	53.16 ± 0.57^cd^	54.45 ± 0.58^d^	52.36 ± 0.17^d^	46.36 ± 0.26^d^	40.20 ± 0.30^c^	43.71 ± 0.85^b^
	**55**	50.18 ± 1.54^de^	50.80 ± 0.20^f^	52.40 ± 0.40^d^	47.12 ± 0.44^c^	42.73 ± 0.38^b^	42.40 ± 0.62^bc^
**206.25**	**440**	68.42 ± 1.02^a^	65.42 ± 0.86^a^	61.63 ± 0.49^a^	47.23 ± 0.29^c^	42.32 ± 0.30^b^	43.39 ± 0.13^bc^
	**220**	66.50 ± 2.41^a^	63.50 ± 1.36^b^	60.72 ± 0.69^a^	47.56 ± 0.37^c^	43.10 ± 0.73^b^	43.24 ± 0.34^b^
	**110**	62.40 ± 1.10^b^	59.40 ± 0.55^c^	56.81 ± 0.36^b^	50.25 ± 0.38^b^	43.30 ± 0.37^b^	45.86 ± 0.40^a^
	**55**	60.15 ± 0.64^b^	58.15 ± 0.25^c^	55.40 ± 0.30^c^	53.65 ± 0.57^a^	45.90 ± 0.62^a^	43.80 ± 0.40^b^

*Means followed by the different lowercase letter in the column of each explant are signifcantly different at *P* < 0.05 according to the Duncan’s Multiple Range Test

At least 20 explant used per experiment

### Determination of chlorophyll and carotenoid

The chlorophyll (chl) and carotenoid contents were extracted using 80% acetone from the fresh micropropagated leaves of *P. khinjuk*. The absorbance of the extracts was measured with a spectrophotometer at 480, 663, and 645 nm. Chlorophyll a, chlorophyll b, and carotenoid contents were calculated with the following equations [18]:

Chlorophyll a: 12.7(A663) – 2.69(A645)

Chlorophyll b: 22.9(A645) – 4.68(A663)

Carotenoid: [A480 +(0.114(A663) – (0.638-A645)]×V/1000×W

### Determination of soluble protein

To determine total soluble protein content, 1 g of fresh leaf samples was homogenized with a chilled pestle and mortar in 5 mL of extraction buffer (0.1 M phosphate buffer, pH 7.0), containing 10 mM KCl, 1 mM MgCl2, 10 mM Na_2_EDTA, and 1% polyvinyl poly pyrrolidone (PVPP), and centrifuged. Next, the supernatant phase was sampled to determine protein content, based on a standard curve prepared with Bovine Serum Albumin (BSA) and expressed as µg g-1 fresh weight [19]. From the fresh plant sample, 0.5 g was homogenized in a 100 mM phosphate buffer (pH 7.0) and centrifuged at + 4 °C and 17,530 g for 20 min. 20 µl of the supernatant, 480 µl distilled water, and 5000 µl Bradford solution were added and the absorbance was measured with a UV-Vis spectrophotometer at a wavelength of 595 nm.

### Determination of shoots fresh weight

Twenty shoots were harvested and immediately weighed on a digital scale with an accuracy of 0.001 g.

### Determination of shoots dry weight

Twenty explant shoots were placed in a paper bag and dried in an oven at 70 °C for 72 h and then weighed.

### Determination of water content of explant (%)

Water content of explant (%): Fresh weight – dry weight / fresh weight × 100.

### Determination of hyperhydric shoots

The shoots that have fragile, translucent stems and curled leaves have been accepted as hyperhydric shoots.

### Determination of hyperhydric shoots rate

Hyperhydric shoots/normal shoots x 100.

### Determination of proliferation rate

Apical and lateral buds that produce new shoots/ Apical and lateral buds that don’t produce new shoots x100.

### Data replication and statistical analysis

The data were assessed by analysis of variance (ANOVA) and the signifcant diferences among mean values (*P* ≤ 0.05) were evaluated by Duncan’s multiple range test (DMRT).

## Results

### Effect of NH_4_NO_3_ and CaCl_2_·2H_2_O on hyperhydricity

**Fig. 1:**
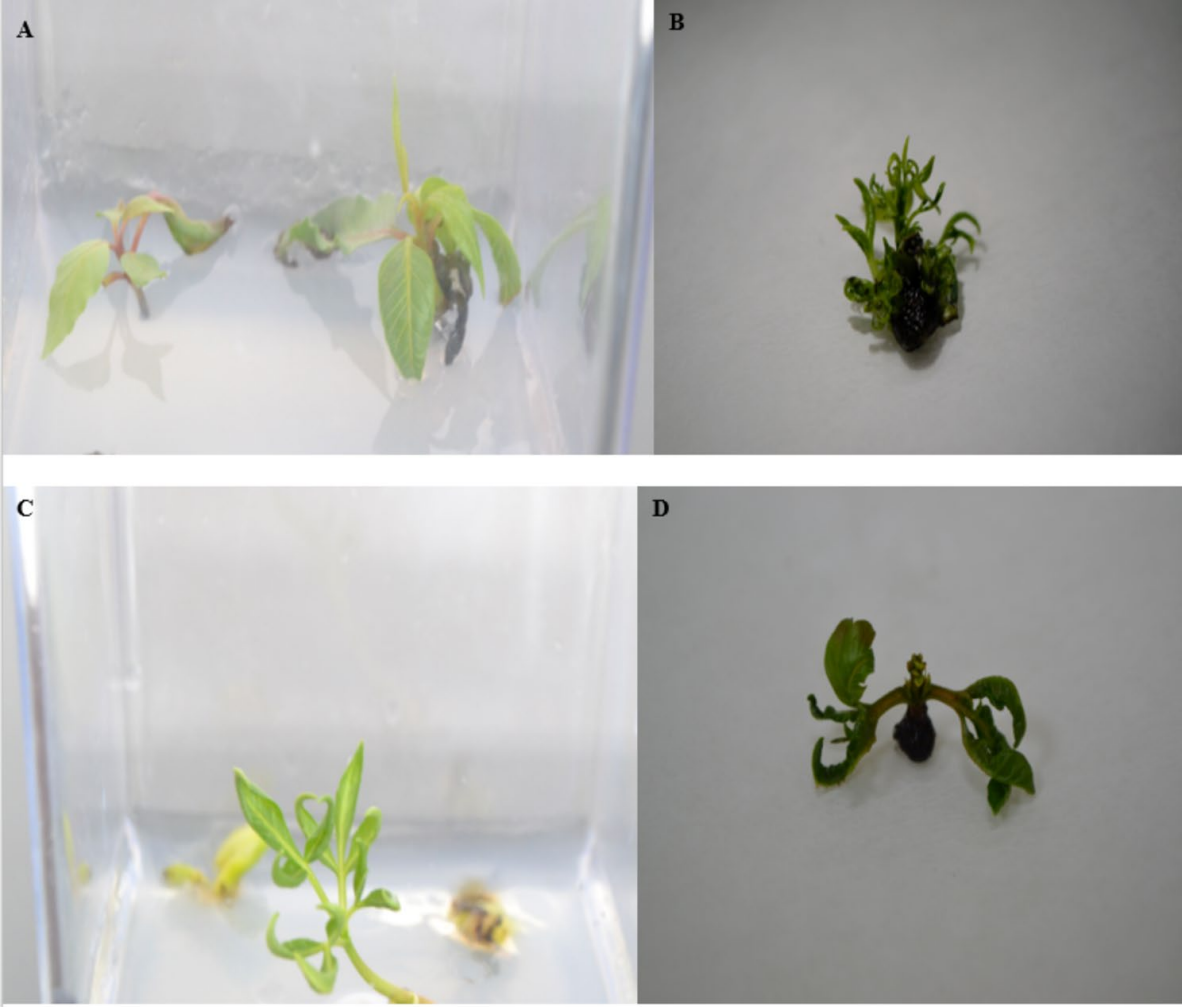
*P. khinjuk* shoots after 28 days of in in vitro incubation. (**A**), Normal shoots (non-hyperhydric). (**B**), semitransparent and fragile, thickened, short internodes stem hyperhydric shoots. (**C**), twisted leaves. (**D**), turgid and brittle leaves

Table 1 was noted that reducing concentrations of NH_4_NO_3_ from 1650 to 206.25 mg/L were resulted in an increase of hyperhydric shoots rate. Symptom of hyperhydric shoots grown with lower concentrations of NH_4_NO_3_ had curled leaves; those grown with higher concentrations of CaCl_2_·2H_2_O had wrinkled leaves and pale green stems (Fig. 1). Table 1 shows that the lowest rate of hyperhydric shoots (34.30%) was grown in the medium containing1650 mg/L NH_4_NO_3_ and 110 mg/L CaCl_2_·2H_2_O. The highest rate of hyperhydric shoots (68.42%) was grown in the medium containing 206.25 mg/L NH_4_NO_3_ and 440 mg/L CaCl_2_·2H_2_O.

### Effect of BAP and mT on hyperhydricity

Table 1 shows that *m*T is far more effective than BAP to reduce hyperhydricity rate. The hyperhydricity rate of *m*T medium were lower than in BAP medium in the all experiments. In addition, reducing concentration of CaCl_2_·2H_2_O from 440 (standard MS medium level) to 110 (modified MS medium level) mg/L can be say a synergistic effect on shoot performance. Since, shoot performance results of 110 mg/L CaCl_2_·2H_2_O medium is higher than 440 CaCl_2_·2H_2_O medium according to the number of shoots per explant, average shoot length and proliferation rate in same type and level of plant growth regulatoars medium (Table 2). The highest value shoots of average number shoots of per explant (2.45), average shoots length (18.47 mm) and proliferation rate (85%) were obtained from the medium containing 110 mg/L CaCl_2_·2H_2_O (Table 2). Shoots characteristics of BAP medium had mostly semitransparent, thickened and short internodes like Fig. 1B. On the other hand, shoots characteristics of *m*T medium had mostly turgid and brittle leaves like Fig. 1D. The highest rate of hyperhydric shoots (68.42%) was obtained from the MS medium containing 206.25 mg/L NH_4_NO_3_, 440 mg/L CaCl_2_·2H_2_O and 0.5 mg/L BAP. The lowest rate of hyperhydric shoots (34.30%) was obtained from the MS media containing 1650 mg/L NH_4_NO_3_, 110 mg/L CaCl_2_·2H_2_O and 1 mg/L *m*T.

**Table 2:** Effect of the combinations of NH_4_NO_3_ and CaCl_2_ ·2H_2_O on shoot performance of *P.khinjuk*

		Average length of shoot*(mm)	Proliferation rate* (%)	Average number of shoots*
Control (Standard MS)	0.5 mg/L BAP	15.10 ± 0.80^d^	65.20 ± 2.13^e^	1.65 ± 0.02^d^
	1 mg/L BAP	15.40 ± 0.41^d^	64.45 ± 2.91^e^	1.80 ± 0.02^abc^
	2 mg/L BAP	16.10 ± 0.60^d^	64.58.±2.17^e^	1.81 ± 0.04^abc^
	0.5 mg/L *m*T	17.42 ± 0.88^abc^	75.40 ± 3.00^bcd^	2.20 ± 0.11^ab^
	1 mg/L *m*T	18.26 ± 0.46^ab^	70.20 ± 2.48^de^	2.15 ± 0.15^ab^
	2 mg/L *m*T	18.45 ± 0.28^a^	70.90 ± 1.16^de^	2.05 ± 0.10^ab^
Modified MS 1650 mg/L NH_4_NO_3_ + 110 mg/L CaCl_2_ ·2H_2_O	0.5 mg/L BAP	16.26 ± 1.11d	75.60 ± 1.78^bcd^	1.82 ± 0.06^abc^
	1 mg/L BAP	17.30 ± 0.20^abc^	78.71 ± 1.85^ab^	1.87 ± 0.09^abc^
	2 mg/L BAP	16.45 ± 0.31^d^	75.63 ± 2.36^bcd^	1.78 ± 0.11^abc^
	0.5 mg/L *m*T	17.38 ± 0.29^ab^	80.30 ± 3.15^ab^	2.30 ± 0.11^ab^
	1 mg/L *m*T	18.47 ± 0.26^a^	85.74 ± 2.78^a^	2.45 ± 0.42^a^
	2 mg/L *m*T	17.61 ± 0.31^ab^	82.20 ± 2.31^ab^	2.35 ± 0.22^a^

*Means followed by the different lowercase letter in the column of each explant are signifcantly different at *P* < 0.05 according to the Duncan’s Multiple Range Test

At least 20 explant used per experiment

### Effect of the combinations of NH_4_NO_3_ and CaCl_2_·_2_H_2_O on shoot propagation

Shoot propagation occurred in all treatments tested. Standard MS medium consisted of the lowest BAP (0.5 mg/L) was produced pale green leaves and shoots stem. Vigorous and high quality shoot was produced on modified MS medium that supplemented with 1 mg/L *m*T. Increasing the concentration of *m*T from 0.5 to 1 mg/L, resulted in an increase the average length of shoot from 17.38 to18.47, average number of shoot from 2.30 to 2.45 and proliferation rate from 80.30 to 85.74 but only on modified medium with *m*T (Table 2). When the results of the number of shoots per explant, average shoot length, and proliferation rate in modified MS medium (1650 mg/L NH_4_NO_3_ and 110 mg/L CaCl_2_·2H_2_O) that caused the lowest hyperhydricity rate and standard MS nutrient medium (1650 mg/L NH_4_NO_3_ and 440 mg/L CaCl_2_·2H_2_O) were compared, the most shoots per explant (2.45), longest average shoot length (18.47 mm) and highest proliferation rate (85%) have been observed in modified MS nutrient medium containing 1 mg/L *m*T (Table 2).

### The analysis of soluble protein

Soluble protein content of normal shoots was higher (2.12 ± 0.38) than hyperhydric shoots (1.66 ± 0.28) (Table 3).

**Table 3:** Total soluble protein, chlorophyll, carotenoid and water content of hyperhydric and non hyperhydric tissues of *P.khinjuk*

	Soluble Total Protein* (mg/ g FW)	Chlorophyll* a (mg/ g FW)	Chlorophyll* b (mg/ g FW)	Carotenoid* (µg /g FW)	Water content* (%)
Normal shoots (non-hyperhydric)	2.12 ± 0.38^a^	0.61 ± 0.56^a^	0.35 ± 0.26^a^	11.75 ± 0.48^b^	78.70^b^
Hyperhydric shoots	1.66 ± 0.28^b^	0.43 ± 0.18^b^	0.25 ± 0.35^b^	18.29 ± 1.25^a^	88.55^a^

*Means followed by the different lowercase letter in the column of each explant are signifcantly different at *P* < 0.05 according to the Duncan’s Multiple Range Test

### The analysis of chlorophyll

Chlorophyll a content of normal shoots (0.61 ± 0.56) was higher than hyperhydric shoots (0.43 ± 0.18) and chlorophyll b content of normal shoots (0.35 ± 0.26) was higher than hyperhydric shoots (0.25 ± 0.35) (Table 3). Hyperhydric shoots were appeared pale green and translucent than normal shoots.

### The analysis of carotenoid

Carotenoid content of hyperhydric shoots (18.29 ± 1.25) were higher than normal shoots (11.75 ± 0.48) (Table 3).

### The analysis of water percentage

Water percentage of hyperhydric shoots (88.55) were higher than normal shoots (78.70) (Table 3). Shoots of high water content had very fragile and thickened stem and leaves.

## Discussion

Recent studies show that the content of NH_4_NO_3_ in the medium affects the level of hyperhydric tissues of in vitro propagated plants [20]. Such as, Jan et al. [21] reported that high levels of NH_4_NO_3_ and KNO_3_ caused a dramatic increase in hyperhydricity rate of *Salvia santolinifolia*. Also in some study, lower concentrations of NH_4_NO_3_ reduce the level of hyperhydricity in different species [22] and hyperhydricity was not observed in MS medium without NH_4_NO_3_ on in vitro shoot of *Brassica oleracea* [23]. A decrease (2–3 times) in the concentration of NH_4_NO_3_ in MS medium reduces the level of hyperhydricity in *Prunus avium* [24] and *Phoenix dactylifera* [24]. Hyperhydricity decrease in plum when using medium with a low NH_4_NO_3_ and KNO_3_ content of MS medium [24]. This study could show a causal link between hyperhydricity and NH_4_NO_3_ level. Since the result of this study confirms recent studies and suggest that an increase in the level of NH_4_NO_3_ from 206.25 to 1650 mg/L can be reduce hyperhydricity rate of micropropagated *P. khinjuk* shoots.

The correlate of the degree of hyperhydric tissue rate on the high concentration of CaCl_2_·2H_2_O in the medium was observed in in vitro cultivated shoots of apple and gerbera [25], it has been shown that the presence of CaCl_2_·2H_2_O in the medium at high concentrations can cause hyperhydricity. Jan et al. [20] reported that high levels of CaCl_2_·2H_2_O in the medium caused a dramatic increase in hyperhydricity rate of *in vitro Salvia santolinifolia* shoots. In addition, Machado et al. [26] reported that high concentrations of CaCl_2_·2H_2_O reduced hyperhydric tissue rate of *Lavandula angustifolia* shoots. Increasing CaCl_2_·2H_2_O concentration is an effective means of reducing hyperhydric tissues in some tree species too [24]. *P. khinjuk* shoots that grown in this study, were grown in the different level of CaCl_2_·2H_2_O medium had not a certain link between hyperhydricity and level of CaCl_2_·2H_2_O. Since significant difference was not observed in hyperhydricity rate of CaCl_2_·2H_2_O concentration from 110 to 440 mg/L. So we could not show a certain corelation between hyperhydricity rate and CaCl_2_·2H_2_O level in *in vitro P. khinjuk* shoots.

Recent studies have been shown that the presence of benzyladenine (BA) in the medium at both low and high concentrations can cause hyperhydricity for carnation, apple and pine [27]. High concentrations of BA induced high hyperhydricity rate of *in vitro Salvia santolinifolia* shoots on standard MS medium [21]. Furthermore, Jan et al. [21] discovered that using BA produced higher percentage of hyperhydricity than 2iP in same study. Also, Liu et al. [28] observed that rate of hyperhydricity of *in vitro Allium sativum* shoots was higher with kinetin than BA. Similarly, Abdouli et al. [15] reported that *m*T was more effective than BA in reducing hyperhydricity rate in *in vitro Pictacia vera* shoots. Replacement of BA with *m*T also led to a decrease in hyperhydricity symptoms in apple and pear shoots [29]. This study shows that increase the concentration of BAP and *m*T from 0.5 to 1 mg/L were reduced the hyperhydricity rate of in vitro shoots of *P. khinjuk* on modified and non modified MS medium, but *m*T was far more effective according to low hyperhydricity rate. This study agrees with previous studies that the type and the density of phytohormone affect the hyperhydricity of in vitro shoots.

Type of explants, culture medium, variety and density of growth regulators, humidity [30], type of gelrite and sucrose density were caused stress on in vitro explants [31]. The function of carotenoids is associated with plant response to environmental stresses [32]. Light, temperature, chilling, drought, and salinity change carotenoid content [24]. Recent studies have indicated that stress factors in culture medium could result in hyperhydric tissue for garlic, sugar beet [24, 26], and *Dendrobium officinale* [33]. This study results showed that the carotenoid content of hyperhydric shoots was significantly higher in hyperhydric shoots than in normal shoots. This may indicate that hyperhydric shoots genetically are very sensitive than normal shoots. Since particular types of plants and even different genotypes of the same plants can have different responses in same in vitro culture conditions [34].

Hyperhydric tissue has inadequate chlorophyll and surplus fluid in the intercellular spaces. This changes enzyme activity and protein synthesis and normal metabolic processes [12]. Type and concentration of cytokinin were caused the low protein content in hyperhydric explants [20] Recent studies show that hyperhydric explants have lower protein contents than normal explants. The results of this study are similar to recent studies since, hyperhydric shoots were found to have lower soluble protein content than normal shoots.

Hyperhydric tissue has multiple deficiencies such as appear turgid, watery at their surface, contain much water in tissue intercellular spaces, less lignification, somewhat transculent, in some cases less green than normal and brittle [14]. This aspect has been reported for in vitro shoots of gerbera [35], carnation [36], shallot [37], *Annona glabra* [38], and *Dendrobium officinale* [34]. Hyperhydric stem and leaves of this study have a pale green appearance. Since chlorophyll a, b content of normal shoots was higher than hyperhydric shoot and water content of hyperhydric shoot was higher than normal shoots.The pale green appearance can orginate from high water and low chlorophyll content of hyperhydric stem and leaves.

## Conclusions

Cytokinin types and it’s concentration and medium modification had an impact on hyperhydricity. Hyperhydricity in shoots of *P. khinjuk* was induced by NH_4_NO_3_, CaCl_2_·2H_2_O, *m*T and BAP. The used low level of NH_4_NO_3_ causes increasing hyperhydric shoots compare of high level. 1650 mg/L NH_4_NO_3_, 110 mg/L CaCl_2_·2H_2_O and 1 mg/L *m*T combination have a synergistic effect on lower hyperhydricity rate than the other combinations that used in this study. Based on the results of this study, it can be concluded that MS medium containing 1650 mg/L NH_4_NO_3_, 110 mg/L CaCl_2_·2H_2_O and 1 mg/L *m*T could be a suitable than standard MS and BAP for the shoots performance (according to shoots of per explant, average shoot length, proliferation rate), lower hyperhydricity rate and shoot quality (according to value of chlorophylla-b, soluble protein and water content) of *in vitro Pistacia khinjuk* shoots.

## Data Availability

Data is provided within the manuscript.

## Abbreviations

Not: applicable
